# First Evidence of Microplastics in the Yolk and Embryos of Common Cuttlefish (*Sepia officinalis*) from the Central Adriatic Sea: Evaluation of Embryo and Hatchling Structural Integrity and Development

**DOI:** 10.3390/ani13010095

**Published:** 2022-12-27

**Authors:** Giulia Chemello, Viola Faraoni, Valentina Notarstefano, Francesca Maradonna, Oliana Carnevali, Giorgia Gioacchini

**Affiliations:** Department of Life and Environmental Science, Polytechnic University of Marche, 60131 Ancona, Italy

**Keywords:** cuttlefish, microplastics, embryonic development, yolk, hatchling

## Abstract

**Simple Summary:**

Microplastic accumulation in aquatic environments could represent a potential risk to wildlife health and survival. Their transfer along the trophic web could threaten ecosystem stability. Over the years, the common cuttlefish (*Sepia officinalis)* has represented an important resource for the fishery of the Adriatic Sea. In this study, for the first time, we assessed the presence of microplastics within cuttlefish eggs, more precisely in embryos and yolk. The presence of microplastics has already been described in common cuttlefish adult specimens. Our findings highlighted how this species is subjected to microplastic exposure starting from early-life stages. The possible effects of microplastic accumulation on embryo health and development should be investigated considering that the reproductive strategy of this species consists of adults laying eggs in separate batches during the single spawning season at the end of which both males and females die. Therefore, population stability primarily depends on the survival and correct development of embryos.

**Abstract:**

Once they reach the aquatic environment, microplastics (MPs) are accidentally ingested by aquatic biota, thus entering the food chain with possible negative effects. The present study investigated, for the first time, MP presence in cuttlefish (*Sepia officinalis*) eggs and their association with embryonic development. Cuttlefish eggs were sampled from four different sites along the Marche region (Senigallia, Ancona, Numana, and San Benedetto del Tronto). Embryo and hatchling biometric parameters were evaluated and the internal structural integrity was examined through histological analysis. MPs were detected and characterized in embryos and yolk samples. MPs were identified in all sites (size < 5 µm), however, their presence has not been associated with an impairment of either embryo or hatchling internal structures. Noteworthy, the highest number of MPs (in both yolk and embryo samples) were found in Numana (37% of the total amount), where the lowest hatchling size was observed. On the other hand, the highest embryo mantle length was associated with the lowest number of MPs detected (9%) in Ancona. Overall, only MP fragments and sphere types (74 and 26%, respectively) were observed, and the most frequent polymers were Polyvinyl chloride (52%), Polypropylene, and Cellulose acetate (15% both). Further studies are needed to assess the possible MP effects on the yolk quality and assimilation.

## 1. Introduction

Over the last 50 years, plastic production has experienced steady and significant growth thanks to its involvement in several industry sectors (e.g., packaging, electronics, transportation, and biology) and consequently, also the number of microplastics (MPs), conventionally defined as small plastic particles (size comprise between 1 µm and 5 mm), has inevitably and rapidly increased [1,2]. Existing MPs comprise both manufactured MPs designed for specific applications (e.g., pellets/powders and engineered plastic microbeads used in cosmetic formulations, cleaning products, and industrial abrasives) and MPs created by the gradual degradation and fragmentation of larger plastic items [3]. Regardless of their origin, MPs are released in different environmental compartments and have spread like wildfire around the globe from land to fresh- and seawater [4,5]. Due to their physicochemical properties, MPs persist in aquatic environments and interact with the aquatic biota at each trophic level. Indeed, biomonitoring assessment studies revealed that plastics and MPs are accidentally ingested by several species of crustaceans, mollusks, fish, marine mammals, and sea birds [6,7,8,9,10]. Toxicity tests conducted in model organisms (e.g., *Danio rerio*) under laboratory conditions highlighted several adverse effects associated with MP exposure and internalization such as larval hypoactivity, intestinal inflammation, oxidative stress, transcriptional changes in the immune response, metabolism disorders, and microbiome alteration [11,12,13,14]. However, MP toxicity is species-specific and depends on both the type of plastic fragments and the abiotic environmental conditions [15].

The tendency of MPs to accumulate and move through the trophic web also implies a potential risk to human health. Many species involved in the bioaccumulation mechanism have a high commercial interest and are consumed daily by humans [16]. Therefore, these species should be investigated with particular attention. The common cuttlefish (*Sepia officinalis)* has always represented an important resource in the fishery history of the Mediterranean Sea [17,18,19]. To date, it is one of the most abundant cephalopod species captured by trawl fishing activities in the Adriatic Sea, especially along its northern and central part extending from the waters off the Friuli Venezia Giulia up to the area off the Abruzzo coast [20]. Despite their relevance in the seafood market, cephalopods are the molluscan class that has received the least consideration regarding MP contamination. Cuttlefish, together with Pacific jumbo squid (*Dosidicus gigas*), and two deep-sea squids (*Vampyroteuthis infernalis* and *Abralia veranyi*), are the only four cephalopod species studied in terms of ingestion and accumulation of plastic fragments [7,10,21,22].

Evidence of MP presence in wild adult specimens of common cuttlefish was demonstrated by Abidli et al. (2019) [10] and by Oliveira and collaborators in 2020 [7], who identified MP fibers, fragments, and microfilm pieces of different polymer types in the digestive tract, with the highest number of fibers detected in the digestive gland of both wild-caught and cultured adult cuttlefish [7]. However, the fate of MPs after reaching the digestive tract in cuttlefish has been only hypothesized [7]. Among plausible options, the most alarming one suggests the MPs’ capacity to cross the epithelium of the digestive tract and potentially enter the hemolymph. A possible target organ could be the ovary, where once reaching the follicles, they may reach the oocyte and consequently accumulate in embryos affecting their development and organogenesis. To our knowledge, neither MP presence in cuttlefish eggs nor its association with embryonic development has been explored. The present study is intended to fill such a gap by investigating the presence of MPs in both embryos and yolks collected from egg batches, evaluating both the biometric parameters and the internal organs and tissues of cuttlefish embryos and hatchlings associated with their MP content. To obtain solid results, the internal structure morphology and functionality of embryos and hatchlings were investigated through histological analysis by applying different staining protocols which highlighted organ compartments and cell types. Concerning the analysis of MP occurrence on biological samples, such as tissue or fluid, the main analytical processes included chemical sample digestion, filtration, and polymer identification by Fourier transform infrared (FTIR) or Raman (RMS) microspectroscopy. Among these, RMS has been widely applied for polymer identification since it offers the most exhaustive information on MP morphological features and their chemical composition in terms of both polymer matrices and pigments [23].

## 2. Materials and Methods

### 2.1. Samples Collection

Cuttlefish eggs were collected, in collaboration with local fishermen, from the fishing gears where trapped females had released their eggs. Samplings were performed within June 2021 from 4 different areas/sites off the coast of the Marche region, site A, from an area off the coast of Senigallia (AN); site B, off the coast of Ancona (AN); site C, off the coast of Numana (AN); and site D, off the coast of San Benedetto del Tronto. Eggs were collected from each sampling site and successively embryos were sampled. Only the latest embryonic stages (28–29) were selected following the identification criteria suggested by Boletzky et al. (2016) [24] based on morphological features (such as eye color and position, arm structure, and chromatophores distribution) and the mantle length (4.7 ± 0.3–5.5 ± 0.4 mm for 28th and 29th stages, respectively). Each embryo selected was weighed and the mantle length was recorded. Yolk samples and whole embryos were collected separately in 3 pools of 10 embryos and 3 pools of 10 yolks weighed and stored in glass tubes at −20 °C for microplastic detection analysis. A total of 10 embryos (between 28–29 stage) per site were also sampled and kept in formaldehyde/glutaraldehyde solution (NaH_2_PO_4_–H_2_O + NaOH + Formaldehyde (36.5%) + Glutaraldehyde (25%) + H_2_O) at 4 °C for 24 h and successively washed in 70% ethanol three times (15 min each) and stored in the same ethanol solution at 4 °C to perform histological analysis.

Due to the different times of spawning among the sampling sites, only in site B (Ancona) and site C (Numana), embryos ready to hatch were identified at sampling time. Therefore, spontaneous non-premature hatchlings from site B and site C were collected during the sampling day and their weight, and mantle length were carefully measured (n = 40 per site), 10 hatchlings per site were stored in formalin solution at 4 °C for 24 h and successively washed and stored in 70% ethanol solution at 4 °C to perform histological analysis.

### 2.2. Ethical Statement

According to Italian legislation (D.L. 04/04/14 N.26 art1 a.1), ethical approval is not required for experiments carried out on vertebrates and cephalopods at embryonic or larval stages that are unable to feed themselves independently

### 2.3. Histological Analysis

Histological analysis was performed following the method described by Chemello et al. [25]. Samples in 70% ethanol were processed to perform the histological analysis. At first, samples were dehydrated in increasing solutions of ethanol (80, 95, and 100%), then washed with xylene (Bio-Optica, Milan, Italy) and embedded in paraffin (Bio-Optica). The paraffin embedding was performed by arranging embryo and hatchling samples to obtain both longitudinal (5 embryos and 5 hatchlings) and transversal (5 embryos and 5 hatchlings) sections for each site. Solidified paraffin blocks were cut into 5 µm sections using a microtome (Leica RM2125 RTS, Nussloch, Germany) and then stained using 3 different staining protocols: Mayer’s haematoxylin and eosin Y stain (Merck KGaA), Alcian blue stain (Bio-Optica) and Masson’s trichrome stain (Bio-Optica). Sections were observed with an optical microscope (Zeiss Axio Imager.A2, Oberkochen, Germany) and images were acquired with a combined color digital camera Axiocam 503 (Zeiss, Oberkochen, Germany).

### 2.4. Microplastic Extraction Protocol

Microplastic extraction was performed at the laboratory of developmental and reproductive biology at the Department of Life and Environmental Science (DISVA) of Polytechnic University of Marche (Ancona, Italy). Yolk and embryo pools were digested in a pre-filtered 10% KOH solution (fiber-glass filter, 1.6 µm pore-size, Whatman GF/A) made with deionized water and KOH tablets (Sigma-Aldrich, Milan, Italy). MP extraction was performed following a modified version of the existing protocol described by Di Renzo et al. (2021) [26] and summarized below. The solution was added to each sample (ratio 1:10 *w*/*v*) and incubated at 40 °C for 24 h. After 24 h, the digestates were filtered in fiber-glass filters (1.6 µm pore-size, Whatman GF/A, Merck KGaA, Darmstadt, Germany) using a vacuum pump connected to a filter funnel. The filter membranes were dried at room temperature and placed into glass Petri dishes until the visual inspection for the identification and characterization of plastic particles.

### 2.5. Microplastics Quantification and Identification

Quantification and identification of microplastics were performed following the method described by Ragusa et al. (2022) [23]. The inspection of filters for the quantitative and qualitative assessment of MPs was performed at the optical microscopy of XploRA Nano Raman Microspectrometer (Horiba Scientific, Rome, Italy) at ×100 magnification. Each filter was inspected moving laterally from the top to the bottom, from left to right, and in the opposite direction to cover the whole filter’s surface. Microparticles that were assumed by eye to be MPs were registered according to number, color, and maximum diameter. Then, Raman Microspectroscopy analysis was performed by using an XploRA Nano Raman Microspectrometer (Horiba Scientific), at the ARI Laboratory of Polytechnic University of Marche (Ancona, Italy). All filter membranes, including the procedural and the environmental blanks, were inspected by visible light using a ×10 objective (Olympus MPLAN10x/0.25) and by using the results of the stereomicroscope as a guide. The thorough morphological characterization was performed by a ×100 objective (Olympus MPLAN100x/0.90) and then analyzed directly on the filter by RMS (spectral range 400–1800 cm^−1^, 532 nm, or 785 nm laser diode). Raw Raman spectra were polynomial baseline-corrected, and vector-normalized (Labspec 6 software, Horiba Scientific). The chemical composition of the detected particles was identified by comparing the collected Raman spectra with several spectral libraries of polymers and pigments [27,28]. Similarities higher than 80 on the Hit Quality Index (HQI) were considered satisfactory.

### 2.6. Quality Assurance and Control (QA/QC)

During sample collection, storage, processing, and analysis efforts were implemented to avoid microplastic contamination. For this purpose, a plastic-free protocol was adopted during all phases of the analysis, and a dedicated room was used for the digestion of samples, filtration, and Raman microspectroscopy analysis steps. Plastic tools were replaced with sterilized glass ones. Cotton laboratory coats and disposable latex gloves were worn during all phases of the sampling and the analysis. All liquids, including ethanol and deionized water for cleaning and preparation of all solutions, were filtered through 1.6 µm pore-size filter membranes (Whatman GF/A). Work surfaces were thoroughly washed with 70% ethanol before starting all procedures. Glassware and instruments, including scissors and tweezers, were washed using dishwashing liquid, triple rinsed with 70% ethanol, and finally rinsed with 1.6 µm filtered deionized water. Moreover, environmental and procedural blanks were prepared and thoroughly analyzed to detect microplastic contamination deriving from the laboratory environment and other external sources. Regarding environmental blanks, a filter membrane soaked with 1.6 µm filtered deionized water was placed into an uncovered Petri dish and positioned each day in the above-mentioned dedicated room. A procedural blank was also prepared together with every batch of samples following the same procedure as samples, without adding yolks or embryos. The filters deriving from environmental and procedural blanks also were first inspected with the optical microscopy of XploRA Nano Raman Microspectrometer (Horiba Scientific) at ×100 magnification.

### 2.7. Statistical Analysis

The statistical analyses were conducted using the GraphPad Software Prism8 for Windows. Means of MPs in each yolk, MPs per g of yolk, MPs in each embryo, number of MPs per g of the embryo, embryos’ dorsal mantel length and weight were analyzed through one-way ANOVA followed by Tukey’s multiple comparisons test, whereas hatchling parameters were analyzed through an unpaired *t*-test (two-tailed) after the normal distribution was verified using the Kolmogorov–Smirnov test. For all the analyses the significance was set at *p* ≤ 0.05.

## 3. Results

### 3.1. Embryos and Hatchlings Biometric Measurements

**Figure 1:**
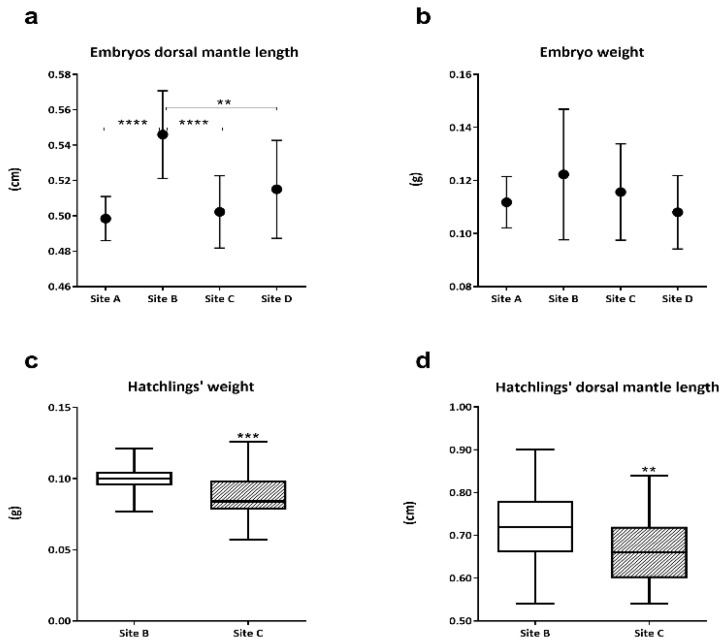
Dorsal mantle length (**a**) and weight (**b**) of Sepia officinalis embryos among sampling sites expressed as mean values ± SD. Boxplot (min. to max.) of hatchlings’ weight (**c**) and dorsal mantel length (**d**) from site B and site C; the line inside boxes represented the mean value. Site A = Senigallia; site B = Ancona; site C = Numana; site D = San Benedetto del Tronto. ** = *p* ≤ 0.01; *** = *p* ≤ 0.001; **** = *p* ≤ 0.0001.

Considering the dorsal mantle length of embryos between the 28 and 29 stages, values from site A, site C, and site D were significantly lower (*p* ≤ 0.0001, *p* ≤ 0.0001, and *p* ≤ 0.01, respectively) than those from site B (Figure 1a). Embryo average weight appeared the highest in site B with respect to the other sampling sites, however, the differences among embryo weights were not significant (*p* > 0.05) due to the high variability of samples analyzed (Figure 1b). The comparison between hatchling weight and mantle length highlighted significantly lower values (*p* ≤ 0.001 and *p* ≤ 0.01, respectively) in animals from site C compared to hatchlings from site B (Figure 1c,d).

### 3.2. Histological Analyses

**Figure 2:**
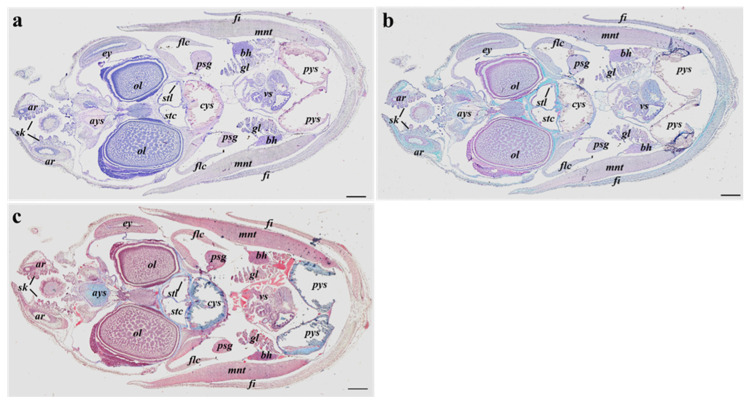
Example of longitudinal sections of *S. officinalis* representative of both embryos at the 28–29 development stages and hatchlings from all sampling sites, stained with Haematoxylin eosin stain (**a**); Alcian blue stain (**b**); Masson’s trichrome stain (**c**); ar, arms; ays, anterior yolk sac; bh, branchial heart; cys, central yolk sac; ey, eye; fi, fins; flc, funnel-locking cartilage apparatus; gl, gills; mnt, mantel; ol, optical lobe; psg, posterior salivary gland; pys, posterior yolk sac; sk, suckers; stc, statocyst; stl, statolith; vs, visceral sac. Scale bar: 500 µm.

The histological analysis did not highlight evident structural or morphological differences among embryos from different sampling sites and between hatchlings from sites B and C. Moreover, the three stains performed showed that all the internal organs and tissues of adult specimens are formed or sketched at the same developmental level in all samples analyzed (Figure 2a–c). In particular, the different staining protocols evinced the same biochemical composition among the anterior, central, and posterior inner yolk sacs.

**Figure 3:**
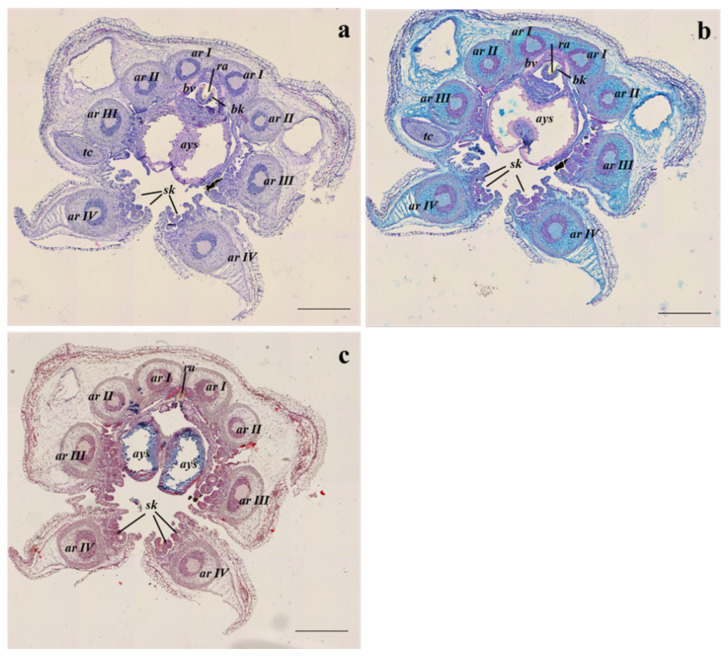
Example of transversal sections of the anterior part at the buccal mass level of *S. officinalis* representative of both embryos at the 28–29 development stages and hatchlings from all sampling sites, stained with (**a**) Haematoxylin-eosin stain; (**b**) Alcian blue stain; (**c**) Masson’s trichrome stain. ar I-IV, arm I-IV; ays, anterior yolk sac; bk, beak; bv, blood vessel; ra, radula; sk, suckers; tc, tentacle. Scale bar: 500 µm.

**Figure 4:**
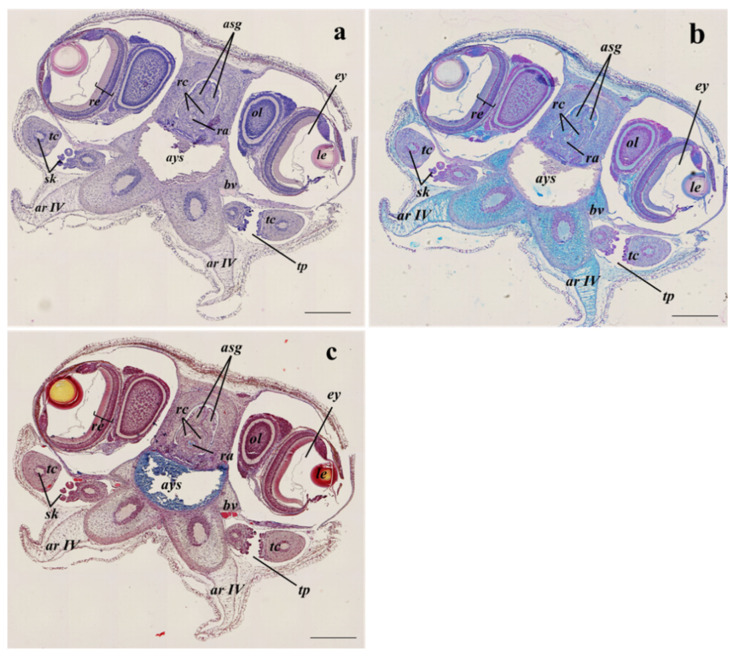
Example of transversal sections of the anterior part at the visual system level of *S. officinalis* representative of both embryos at the 28–29 development stages and hatchlings, from all sampling sites, stained with Haematoxylin-eosin stain (**a**); Alcian blue stain (**b**); Masson’s trichrome stain (**c**). ar IV, arm IV; asg, anterior salivary gland; ays, anterior yolk sac; bv, blood vessel; ey, eye; le, lens; ol, optical lobe; ra, radula; rc, radula cartilage; re, retina; sk, sucker; tc, tentacle; tp, tentacular pocket. Scale bar: 500 µm.

Following the anterior–posterior axis, the two formed tentacles equipped with suckers (Figure 3a–c) and some components of the buccal complex such as the radula, surrounded by the radula cartilage, the beak, and the anterior salivary gland (Figure 4a–c) can be observed. The ocular lobules and the eyes are well developed, they are located slightly dorsal to the head, and they are protected by poorly developed outer lids (Figure 4a–c).

**Figure 5:**
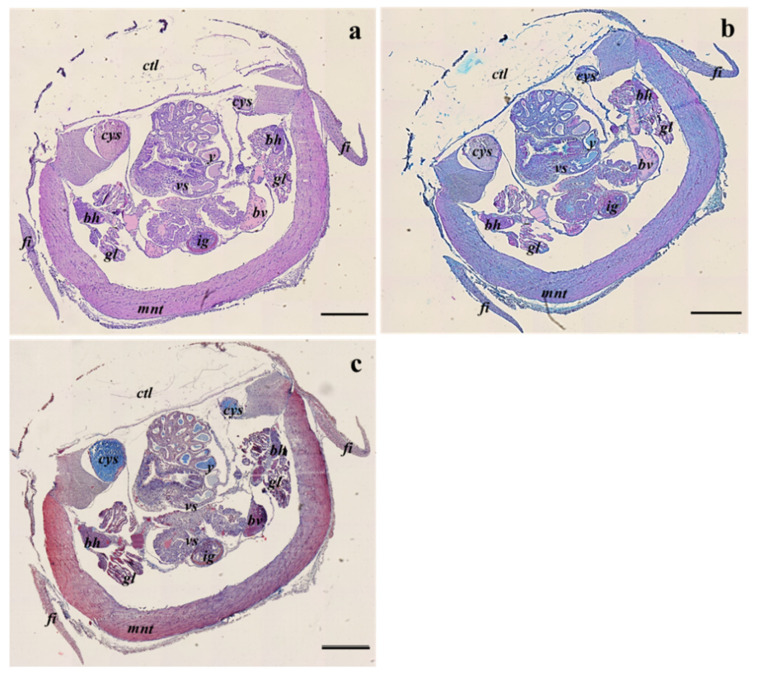
Example of transversal sections of the central part at the visceral sac level of *S. officinalis* representative of both embryos at the 28–29 development stages and hatchlings, from all sampling sites, stained with Haematoxylin-eosin stain (**a**), Alcian blue stain (**b**), and Masson’s trichrome stain (**c**). Bh, branchial heart; bv, blood vessel; ctl, cuttlebone; cys, central yolk sac; fi, fins; gl, gills; ig, ink gland; mnt, mantle; vs, visceral sac; y, yolk. Scale bar: 500 µm.

In the central body part, the complexity of the internal structure increases ventrally to the mantle cavity within the pallial cavity delimited by the mantle (Figure 5a–c). The digestive structures are fully formed within the visceral sac although the digestive gland is not fully functional. The visceral sac comprises also the central inner yolk sac, gills and gill hearts, the functional ink gland, and the ink sac (Figure 5a–c).

### 3.3. Microplastics Quantification and Identification

#### 3.3.1. MP Detection in Yolk and Embryos in Each Sampling Site

Raman microspectroscopy analysis identified a different number of MPs in yolk and embryo samples among the sampling sites; all MPs sizes were <5 µm. In all sampling sites considered, a higher number of MPs were found in yolk samples compared to embryo pools (Table 1). Site B (Ancona) was the only site with no MPs detected in embryos and the site with the lowest number of MPs observed. The two sampling sites with the highest number of MPs per sample’s weight in both yolks and embryos were sites A and C.

#### 3.3.2. MP Polymers Identification in Yolk and Embryos in Each Sampling Site

The identification of polymer types showed that Polypropylene (PP) and Polyvinyl chloride (PVC) were the two most frequent polymers among sampling sites (Table 2).

#### 3.3.3. Total Amount of Microplastics and Distribution of Polymers and Colors among Sampling Sites

**Figure 6:**
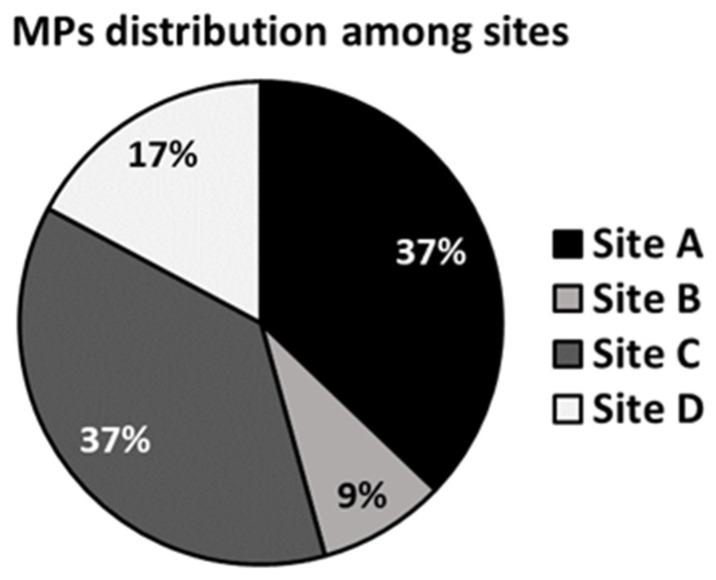
Total microplastics percentage detected in each sampling site. Site A = Senigallia; site B = Ancona; site C = Numana; site D = San Benedetto del Tronto.

**Figure 7:**
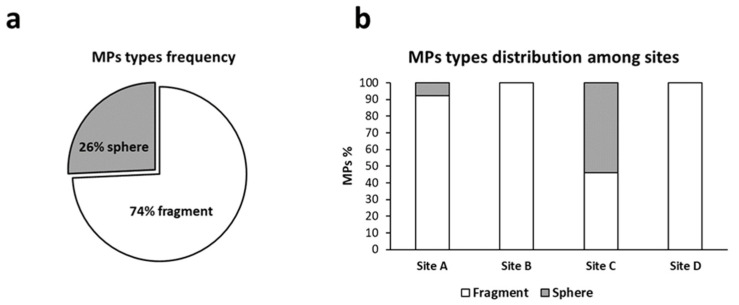
Percentage frequency of MP types (**a**) and types distribution among sampling sites (**b**). Site A = Senigallia; site B = Ancona; site C = Numana; site D = San Benedetto del Tronto.

The sites with the highest number of MPs were Site A and C which both represented 37% of the total amount (Figure 6). Only two MP types were detected among all the samples observed: fragments, corresponding to most MPs (74%), and spheres (26%) (Figure 7a). MP fragments and spheres were observed only in sites A and C, whereas in sites B and site D only MP fragments were detected (Figure 7b).

**Figure 8:**
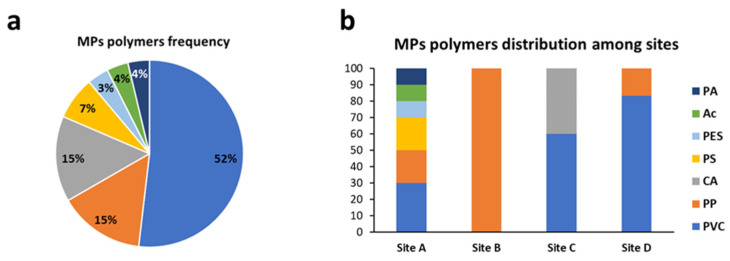
(**a**) Percentage frequency of MP polymers and their distribution (**b**) among sampling sites. PVC, polyvinyl chloride; PP, polypropylene; CA, cellulose acetate; PS, polystyrene; PES, polyethersulfone; AC, acrylic; PA, polyamide. Site A = Senigallia; site B = Ancona; site C = Numana; site D = San Benedetto del Tronto.

**Figure 9:**
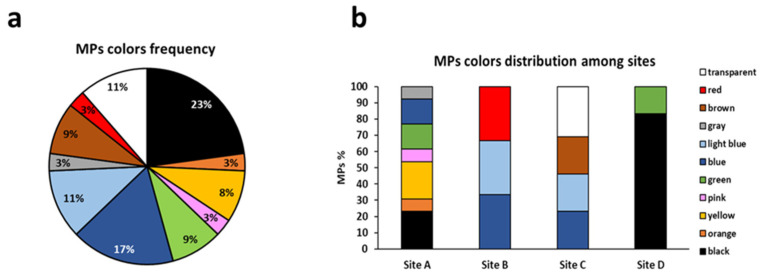
Percentage frequency of MP colors (**a**) and color distribution among sampling sites (**b**).

The most frequent polymer identified was PVC which accounts for more than half of MPs observed (52%) followed by PP and CA (both 15%) (Figure 8a). The site with the highest number of polymers is site A whereas in site B only PP was observed (Figure 8b). Regarding the MP colors, a high variety of colors was registered. The most frequent colors were black, blue, transparent, and light blue, representing 23%, 17%, 11%, and 11%, respectively (Figure 9a). No evident trend of colors distribution was observed among the sampling sites, the site with the highest number of colors detected was site A (Figure 9b).

## 4. Discussion

As an intermittent terminal spawner, the common cuttlefish lays the eggs in separate batches during the single spawning season at the end of which both males and females die [29]. Considering its reproductive strategy and short life cycle, population stability primarily depends on successful recruitment, which in turn requires both sufficient breeding rates and embryo survival [30]. The latter is strongly influenced by the variation of several environmental factors including temperature, salinity, light intensity, photoperiod, oxygen saturation, predation, and of course pollution [31]. The present study revealed, for the first time, the presence of MPs in both embryos and yolk samples of common cuttlefish retrieved in four different sites along the Marche region coast. Noteworthy, MP occurrence inside cuttlefish embryos was evidenced in all four sites investigated, but several differences in terms of density and polymers have been highlighted among sites. The exact pathway that leads to MP accumulation in both yolk and embryos is yet to be clarified considering the peculiar structure of *Sepia officinalis* eggs. Cuttlefish eggs are surrounded by the eggshell capsule, a multilayer envelope secreted in synergy by the female oviductal gland, nidamental gland, and ink sac which acts as a protective barrier with limited permeabilization to gases and other chemicals (e.g., trace elements) [32]. During organogenesis, which represents the last phase of embryonic development, the egg capsule becomes permeable allowing the entrance of water and solutes within the egg [33]. MPs suspended in the aquatic environment could pass through the egg capsule and thus reach the ooplasm. However, it should be considered that before the organogenesis, at the end of gastrulation, the yolk is covered by the yolk syncytium and the extra-embryonic ectoderm [23]. This process did not justify the presence of MPs in the yolk samples analyzed in the present study since the yolk should be isolated from the perivitelline space. A second hypothesis could consist of the internalization of MPs by the females and their subsequent transfer to the oocytes before egg deposition. The synthesis of yolk protein precursors (vitellogenin) has been demonstrated to be synthesized mainly by follicle cells in cephalopods [34]. In *Sepia officinalis*, during vitellogenesis, follicular cells are organized as strings that enter the oocytes and synthesize the vitellogenin that will enter the oocyte [35]. The possibility of MPs reaching the ovary should be investigated, considering the transfer of nutrients from the digestive glands and their internalization in the eggs. This organ is analogous to the vertebrate liver and has an active role in digestion (enzyme secretion, absorption of molecules, and nutrient and lipid storage) and detoxification (Costa et al., 2014) [36]. Therefore, the digestive gland could represent the reserve of lipids used by the follicle cell to synthesize the vitellogenin and the target organ of MPs. Indeed, the preferential accumulation of MPs in the cuttlefish digestive gland has been previously demonstrated by Oliveira et al. (2020) and Abidli et al. (2019) [7,10]. Based on this evidence, studies regarding the pathways of lipids stored in the digestive gland and their transfer, together with vitellogenin accumulation into the oocytes are necessary to investigate the fate of MPs once they are accidentally internalized. The cross-generation transfer of plastic fragments has been already demonstrated for nanoplastics (NPs) in zebrafish (*Danio rerio*) [37]. These authors found polystyrene NPs in the yolk sac of zebrafish embryos from females previously fed an NP-containing diet. The presence of polystyrene (PS) NPs in the embryo yolk sac suggested that the yolk is a potential target for their accumulation in adult females and could represent the main route of cross-generation contamination [37]. Although the above-mentioned study considered smaller-size particles (mean diameter 42 nm) than those found in this study (<5 µm), it should be considered that cuttlefish eggs and embryos present larger dimensions than zebrafish and MPs lower than 5 µm could be possibly carried through the bloodstream to reach different target organs [26,38,39]. Moreover, concerning the high variety of MP polymers, shapes, and colors observed, interesting results emerged. The most frequent polymers (PVC, PP, CA, and PS) identified in the present study are some of the most abundant MP types found in the Mediterranean Sea [40,41]. Based on their density, these MPs are differently distributed along the water column, polymers with lower density than seawater as PE, PP, and PS float at the surface, whereas those with higher density (e.g., PVC) tend to sink [42,43]. The common cuttlefish is a nekto-benthic species that lives and preys predominantly on sandy and muddy bottoms from 2–3 m depth along the coastline to approximately 200 m depth and thus should be principally exposed to denser polymers. Nevertheless, low-density MPs could be accidentally ingested by cuttlefish while preying on MP carrier animals such as small pelagic fish and bivalves. Moreover, degradation and fragmentation phenomena plus additives leaching can affect the density of MPs and their distribution along the water column becoming available to a higher variety of species [42]. The unpredictable fate of MPs in the aquatic environment could explain the different frequencies of both polymers and colors observed among the different sampling sites. Interestingly, within the MP shapes identified, no fibers were observed, although they have been reported as the most frequent MP types ingested by marine animals [44]. The absence of MP fibers in cuttlefish embryos and yolk is probably related to their usually large size which represents a limitation to their transfer within the biological compartments. Fibers could be easily ingested by cuttlefish females, although their binding with some carrier to the oocyte and their internalization through endocytosis are practically not plausible.

Focusing on MP density analyses, worthy of note is that the highest number of MPs (in both yolk and embryo samples) was found in site C where the lowest hatchling size was observed. In site B, the highest embryo mantle length was associated with the lowest number of MPs detected. Beyond the variability of these parameters, embryonic development also depends to a large extent on the amount and quality of the yolk sac as it represents the only food source for the embryo, supplying energy and structural components required for optimal embryonic and post-embryonic development [45]. The yolk is divided into two compartments, the outer yolk sac which is absorbed and partially stored in the inner yolk sac [46]. In addition to its role as a nutrients source and reservoir, the outer yolk is also involved in respiratory and circulatory processes during the early phases of organogenesis, acting as a pump in the primary circulatory system until the complete formation of both hearts and gills [46]. Therefore, MPs identified in the outer yolk samples could interfere with yolk resorption and in turn with these yolk-mediated processes and thus represents a potential threat to embryonic correct growth. The strict connection between the outer yolk sac and the embryo also justifies the presence of MPs in embryo samples, suggesting the internalization of these plastic fragments from the outer yolk. However, the target sites of internalized MPs are not clear since part of the outer yolk is absorbed through the yolk syncytium which releases the nutrients into the bloodstream while a portion is directly stored in the inner yolk sac [47].

Finally, despite the occurrence of MPs in embryos from all the investigated sites and their association with embryonic growth, the histological investigation revealed that there are no alterations in the morphology and functionality of the embryonic internal organs and tissues. This result highlights the fact that although there are MPs accumulated inside the embryos, these do not interfere with organogenesis. Although MP presence was not associated with any impairment of internal structures in both embryos and hatchlings, their persistence could affect the life of juveniles and adult specimens.

## 5. Conclusions

In this study, the presence of MPs in *Sepia officinalis* embryos from different sites of the central Adriatic Sea was observed for the first time. These preliminary results could suggest that the presence of MPs does not impact embryo development and organogenesis. However, regardless of the pathway through which MPs enter the egg, a better understanding of their relationship with the embryo and hatchling health and growth is required since these species will experience chronic exposure to MPs from the embryonic development to the adult phase. Further studies are in process to assess the possible MPs effects on the yolk quality and assimilation.

## Figures and Tables

**Table 1:** Number of microplastics detected in *S. officinalis* yolk and embryo pools. Site A = Senigallia; site B = Ancona; site C = Numana; site D = San Benedetto del Tronto; # MPs/yolk, average number of MPs in each yolk; MPs/yolk, average number of MPs per g of MPs/embryo, average number of MPs in each embryo; MPs/embryo, average number of MPs per g of embryo. Values are reported as mean ± standard deviation. Different letters indicate a statistically significant difference (*p* < 0.05).

Site	#Mps/Yolk	MPs/Yolk (MPs/g)	#MPs/Embryo	MPs/Embryo (MPs/g)
A	0.45 ± 0.07 ^a^	14.17 ± 2.33 ^a^	0.2 ± 0.03 ^a^	2.35 ± 0.18 ^a^
B	0.15 ± 0.07 ^b^	4.99 ± 2.13 ^b^	0.00 ± 0.00 ^b^	0.00 ± 0.00 ^b^
C	0.40 ± 0.01 ^a^	18.64 ± 0.18 ^a^	0.25 ± 0.07 ^a^	2.35 ± 0.67 ^a^
D	0.25 ± 0.07 ^ab^	10.59 ± 0.78 ^c^	0.05 ± 0.07 ^b^	0.62 ± 0.88 ^ab^

**Table 2:** Characterization of microplastic polymer types detected in *S. officinalis* yolk and embryo pools. A = Senigallia, B = Ancona, C = Numana, D = San Benedetto del Tronto. CA, cellulose acetate; PA, polyamide; PES, polyether sulfone; Ac, acrylic; PP, polypropylene; PS, polystyrene; PVC, polyvinyl chloride; ND, not detected.

Site	Polymer (Yolk)	Polymer (Embryo)
A	PES, PVC, PA, PP	Ac, PP, PS
B	PP	ND
C	CA, PVC	PVC
D	PVC	PP

## Data Availability

The data presented in this study are available on request from the corresponding author. The data are not publicly available due to privacy reasons.
